# Evolution of Complexity of Palliative Care Needs and Patient Profiles According to the PALCOM Scale (Part Two): Pooled Analysis of the Cohorts for the Development and Validation of the PALCOM Scale in Advanced Cancer Patients

**DOI:** 10.3390/cancers16091744

**Published:** 2024-04-29

**Authors:** Albert Tuca, Margarita Viladot, Gemma Carrera, Lucia Llavata, Carmen Barrera, Manoli Chicote, Javier Marco-Hernández, Joan Padrosa, Carles Zamora-Martínez, Ignacio Grafia, Anais Pascual, Carme Font, Elena Font

**Affiliations:** 1 Unit of Supportive Care in Cancer, Medical Oncology Department, Hospital Clinic and Translational Genomics and Targeted Therapies in Solid Tumors, IDIBAPS, University of Barcelona, 08036 Barcelona, Spain; viladot@clinic.cat (M.V.); gecarrera@clinic.cat (G.C.); llavata@clinic.cat (L.L.); mbarrera@clinic.cat (C.B.); mchicote@clinic.cat (M.C.); jmarco@clinic.cat (J.M.-H.); padrosa@clinic.cat (J.P.); czamora@clinic.cat (C.Z.-M.); grafia@clinic.cat (I.G.); alpascual@clinic.cat (A.P.); cfonf@clinic.cat (C.F.); efont@clinic.cat (E.F.); 2 Psychosocial Support Team, “La Caixa” Foundation (EAPS), Clinic Hospital of Barcelona, 08036 Barcelona, Spain; 3 Chair of Palliative Care, University of Barcelona, 08036 Barcelona, Spain

**Keywords:** early palliative care, advanced cancer, complexity of care needs, integration of palliative care in oncology, specialist palliative care, predictive model of complexity

## Abstract

**Simple Summary:**

The World Health Organisation (WHO) has urged all health organizations to develop programs specifically aimed at integrating palliative care (PC) into existing services, based on a model of shared care from the time of diagnosis and alongside life-prolonging treatments. In a context where the resources of multidisciplinary teams specialized in early palliative care (EPC) are not unlimited, it is very important to reach a consensus on appropriate referral criteria so that all patients who need it receive adequate support in terms of quality and intensity, and that specialized resources are not disproportionately used for those cases with less need. Therefore, in the shared care model proposed by the WHO, identifying the complexity of PC needs is a key aspect in defining the appropriate referral criteria. The PALCOM scale is a five-domain multidimensional assessment tool specifically designed and validated to identify the complexity of the needs of patients with advanced cancer. The study we now present, based on pooled data from the development and validation cohorts, confirms the high predictive ability of the PALCOM scale to identify the level of complexity of needs. The data from this study show that higher levels of complexity are significantly associated with greater instability, healthcare resource use and mortality. This study also highlights the importance of identifying the complexity profiles to optimize the targeted referral and management of the intervention intensity by EPC teams.

**Abstract:**

Introduction: Identifying the complexity of palliative care needs is a key aspect of referral to specialized multidisciplinary early palliative care (EPC) teams. The PALCOM scale is an instrument consisting of five multidimensional assessment domains developed in 2018 and validated in 2023 to identify the level of complexity in patients with advanced cancer. (1) Objectives: The main objective of this study was to determine the degree of instability (likelihood of level change or death), health resource consumption and the survival of patients according to the level of palliative complexity assigned at the baseline visit during a 6-month follow-up. (2) Method: An observational, prospective, multicenter study was conducted using pooled data from the development and validation cohort of the PALCOM scale. The main outcome variables were as follows: (a) instability ratio (IR), defined as the probability of level change or death; (b) emergency department visits; (c) days of hospitalization; (d) hospital death; (e) survival. All the variables were analyzed monthly according to the level of complexity assigned at the baseline visit. (3) Results: A total of 607 patients with advanced cancer were enrolled. According to the PALCOM scale, 20% of patients were classified as low complexity, 50% as medium and 30% as high complexity. The overall IR was 45% in the low complexity group, 68% in the medium complexity group and 78% in the high complexity group (*p* < 0.001). No significant differences in mean monthly emergency department visits (0.2 visits/ patient/month) were observed between the different levels of complexity. The mean number of days spent in hospital per month was 1.5 in the low complexity group, 1.8 in the medium complexity group and 3.2 in the high complexity group (*p* < 0.001). The likelihood of in-hospital death was significantly higher in the high complexity group (29%) compared to the medium (16%) and low (8%) complexity groups (*p* < 0.001). Six-month survival was significantly lower in the high complexity group (24%) compared to the medium (37%) and low (57%) complexity groups (*p* < 0.001). Conclusion: According to the PALCOM scale, more complex cases are associated with greater instability and use of hospital resources and lower survival. The data also confirm that the PALCOM scale is a consistent and useful tool for describing complexity profiles, targeting referrals to the EPC and managing the intensity of shared care.

## 1. Introduction

The World Health Organisation (WHO) has advised about the progressive aging of the population, the global rise in the prevalence of advanced chronic diseases that threaten life and the growing need for palliative care (PC). The WHO urges all healthcare organizations to design especially aimed at integrating PC into existing services based on a shared care model from the diagnosis of the disease and concomitant with treatments to prolong life [1,2].

Multiple controlled clinical trials have demonstrated that early palliative care (EPC) significantly improves the control of symptoms, emotional distress, the perceived quality of life and the satisfaction of patients with advanced cancer [3,4,5,6,7,8,9,10,11,12,13,14,15]. Some of these clinical trials have also confirmed that EPC can increase patient survival, reduce the futile use of chemotherapy and optimize the use of healthcare resources [1,15,16].

Taking into account the position of the WHO and the robust scientific evidence available, most of the scientific societies and consensus documents of experts in cancer recommend care by specialized multidisciplinary EPC teams for all patients with advanced cancer in parallel with etiological treatment (American Society of Clinical Oncology [ASCO], European Society of Medical Oncology [ESMO], National Comprehensive Cancer Network [NCCN], Lancet Commission) [16,17,18,19,20,21]. However, some authors and consensus documents argue that systematic referral of all advanced cancer patients to specialized EPC teams is not always desirable or sustainable due to the limitation of resources in real clinical practice. They point out the urgent need to ensure basic training in PC for all the professionals who attend to patients with advanced cancer at all levels of healthcare (hospital, community), with the aim of reserving the services of specialized EPC teams for situations that go beyond basic training [22,23,24,25]. The basic level of PC should include sufficient training on basic multidimensional evaluation, control of symptoms, shared decision making, advanced planning and management of resources and should be carried out by non-specialized teams (primary PC) or teams specialized in cancer (secondary PC). The specialized level of PC involves advanced and accredited training in the care of patients with higher intensity needs (high symptom burden, refractory pain, severe psychological or spiritual distress, loss of purpose in life, socio-familial risk, conflict in shared decision making, etc.) and is carried out by specialized, hospital or community EPC teams (tertiary PC) based on a shared care model with reference teams [25]. According to these considerations, derivation to EPC and the management of shared care with the primary care and oncology teams depends not only on the extension and prognosis of the cancer but also on the complexity of the PC needs of the patient. The key issue is, therefore, no longer to recognize the effectiveness of EPC teams but to define a referral model that guarantees access to high-quality PC for all patients who need it.

Three models of referral to EPC have been proposed: systematic, on-demand and targeted. Systematic referral proposes care by EPC teams for all patients with advanced cancer following diagnosis [21]. The problem with systematic referral, the model used in most controlled clinical trials, is that it needs a wide network of EPC, which is not always available in real clinical practice, and that it often provides a disproportionate intensity of palliative intervention to patients with low complexity. Referral on demand depends on the sensitivity and clinical criteria of the referring professional, which is often heterogeneous and little consistent. The result is usually a late referral, which reduces the possibility of planned PC adjusted to the specific needs of the patient. At present, the model considered optimal by most authors is targeted referral. It is based on the consensus of the criteria of referral to EPC, which prioritizes early care for patients with PC needs of greater complexity [26,27,28].

The intrinsic difficulty of a targeted referral model, which is easy to accept from a theoretical point of view, is that the categorization of the complexity of PC needs is neither agreed upon nor well-defined. A recent review of the literature identified six models of classification of the complexity of PC needs, aimed at managing referral and shared care with the specialized EPC teams (Hex-Com, Perroca-Scale, AN-SNAP, Hui-Major-Criteria, IDC-Pal, PALCOM) [29,30,31,32,33,34,35,36,37,38,39,40,41,42,43,44,45,46]. The use of these tools allows the complexity of PC needs to be categorized as low, in which basic PC would be indicated (primary or secondary PC) or medium-high, in which care by specialized EPC teams would be systematically indicated (tertiary PC).

The PALCOM scale is a predictive model of the complexity of PC needs that was specifically developed for patients with advanced cancer in 2018 [31,37]. It is a tool composed of a multidimensional scale with five domains of evaluation (symptom burden, refractory pain, functional impairment, socio-familial risk and spiritual/existential problems), which allows classifying, with high sensitivity and specificity, patients as low, medium and high complexity in relation to PC needs. The PALCOM scale is shown in Table 1. The external validation of the PALCOM scale, published in 2023, which confirmed the accuracy of the tool, was the first part of this research project [45]. In the development [31] and validation [45] cohorts of the PALCOM scale, the variables that discriminate the level of complexity at the patient’s initial visit were identified, but the evolutionary behavior of the model during follow-up was not analyzed. We now present the second part of this research, which describes the evolutionary behavior of the complexity of palliative care needs.

Complex systems are characterized by depending on the continuous interaction of multiple variables within an unstable equilibrium that is very sensitive to rapid, and not always predictable, changes from the initial conditions [37,38,39,40,41,42,43,44,45,46]. In accordance with this argument, the analysis of evolutionary instability is essential to understanding the model of palliative complexity.

The present study involves an analysis of the evolutionary behavior of palliative care complexity based on pooled data from the cohorts of the development and validation of the PALCOM scale. The hypothesis is that the levels of greatest complexity present an evolution characterized by greater instability, resource consumption and mortality.

## 2. Materials and Methods

This was a prospective, observational, multicenter study with a longitudinal follow-up of 6 months based on the pooled data of the development and validation cohorts of the PALCOM scale in patients with advanced cancer. Part one of this project was the external validation of the PALCOM scale [46], and part two, which we now present, is the pooled analysis of the development and validation cohorts and also the analysis of the evolutionary behavior of palliative complexity during patient follow-up.

### 2.1. Study Site and Period

**Figure 1:**
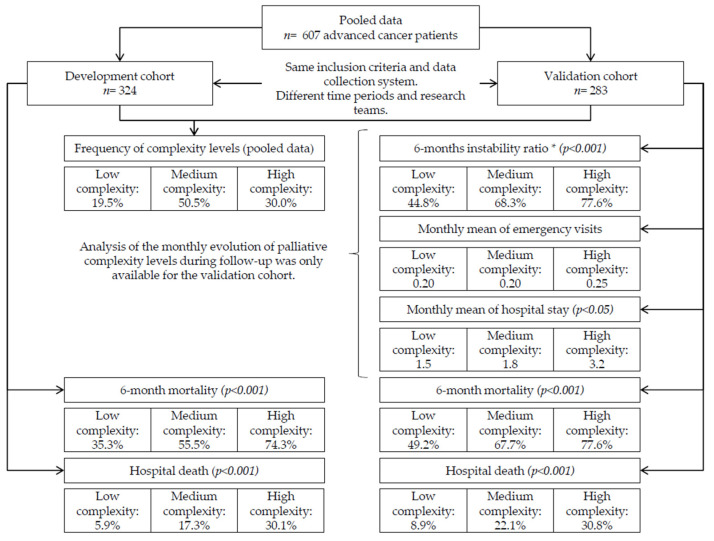
Flow-diagram of pooled data of the PALCOM development and validation cohorts. * Instability ratio: probability of change in the initial condition resulting in an increase in level or death.

Multiple public healthcare centers of all levels of care (primary care, home care, hospital care, medium-long stay units) from the Autonomous Community of Catalunya (Spain) participated in the study. Two consecutive cohorts were studied with the same methodology and data collection system in two different periods and by intentionally different field research teams; the first was the cohort of scale development (November 2012–January 2013) and the second was the validation cohort (December 2020–April 2021). Figure 1 shows a flow chart.

### 2.2. PALCOM Scale of the Complexity of Palliative Care Needs

The PALCOM scale was developed in 2018 [31] and validated in 2023 [46] specifically for patients with advanced cancer. It is a multidimensional scale with five domains of evaluation: (a) symptom burden; (b) refractory pain; (c) functional impairment; (d) socio-familial risk factors; (e) spiritual/existential problems (Table 1). Each domain is dichotomously scored (0 = absence and 1 = presence), with the final result being the total sum of these values (0–5). This tool classifies the complexity of PC needs into three levels: (1) low (score 0–1), in which basic, non-specialized, PC is recommended (primary or secondary PC); (2) Medium (score 2–3), in which shared care with specialized EPC teams is recommended (tertiary PC); (3) high (score 4–5), in which intensive shared care with specialized EPC teams is recommended (tertiary PC).

### 2.3. Study Objectives

The main objective of this study was to identify the grade of evolutionary instability of the levels of complexity of the PC needs assigned in the basal visit and their impact on the consumption of healthcare resources and survival. The secondary objective was to confirm the consistency of the PALCOM model of complexity by comparison of the frequencies of the levels of complexity and domains of the scale affected between the two cohorts.

### 2.4. Inclusion Criteria

It was proposed that all the patients fulfilling the following inclusion criteria be consecutively included in the study: age ≥ 18 years, diagnosis of advanced cancer, clinically estimated life expectancy ≤ 6 months, and signed informed consent.

### 2.5. Main Outcome Variables

In this study, the main outcome variables were as follows: (a) instability ratio of the level of complexity; (b) consumption of healthcare resources; and (c) survival. Instability ratio (IR): Unstable equilibrium is a defining characteristic of complex systems. In this study, the main variable related to this concept was the IR, defined as the monthly probability of presenting a change in the level of complexity or death according to the level of complexity assigned at the baseline visit. Resource consumption: Monthly emergency department use, monthly hospital stay and in-hospital death were analyzed according to the level of complexity assigned at the baseline visit. Survival: Actuarial survival was analyzed according to the level of complexity assigned at the baseline visit.

### 2.6. Descriptive Variables

The following sociodemographic and clinical characteristics of the patients included in the study were analyzed: age, sex, the primary origin of the cancer and domains of the PALCOM scale (high symptom burden, defined as the presence of more than five symptoms of at least moderate intensity in a systematic registry of 10 symptoms based on the Edmonton Symptom Assessment Scale model [47]; potentially refractory pain, according to the Edmonton Classification System for Cancer Pain [ECSCP] [48,49,50]; Karnofsky index; factors of socio-familial risk; ethical/existential/spiritual conflict according to the established classification in the development and validation cohorts of the PALCOM scale) [31,46].

### 2.7. Statistical Method

In both cohort studies, the estimation of the sample size was based on not only previous studies [31,51] but also on the maximum recruitment capacity of the participating centers considering that the variables should be registered in the conditions of their daily clinical practice. For the main objective, the IR, resource consumption and survival stratified by the level of PC complexity assigned in the basal visit were analyzed, while for the secondary objective, the frequencies of the levels of complexity and the domains of the PALCOM scale observed in the development and validation cohorts were compared. Categorical or dichotomic variables were analyzed using absolute and relative frequencies. Continuous variables are described by calculating the mean value and standard deviation (SD) with a 95% confidence interval (95% CI). For the comparison of variables according to the levels of complexity, the Fisher exact test and the non-parametric Mann–Whitney–Wilcoxon tests were used. The function of survival was analyzed with the Kaplan–Meier method. For comparison between the different levels of complexity, we used the stratified log-rank test and hazard ratios (HR) (95% CI) extracted from the Cox model.

## 3. Results

A total of 607 patients with advanced cancer participated in the study, 324 (53.4%) of whom corresponded to the PALCOM development cohort and 283 (46.6%) to the validation cohort. The data of the evolutionary behavior of the model of complexity only corresponded to the validation cohort since these variables were not analyzed monthly in the development cohort. A flow diagram is shown in Figure 1.

### 3.1. Pooled Analysis of the Two Cohorts

Of the 607 patients with advanced cancer included in the two cohorts, 350 (57.7%) were men. The mean age was 70 years (SD ± 59–80) and 355 patients (58.5%) were included in the hospital and 255 (41.5%) in community health centres. The most frequent primary origin of the cancer was the lungs (23.1%), followed by the colon (15.5%), prostate (7.6%), pancreas (7.6%) and breasts (6.8%). The extent of cancer was metastatic in 500 patients (82.4%) and locoregionally advanced in 107 patients (17.6%), and 462 patients (76.1%) were receiving cancer treatment at study entry. The symptoms most frequently observed in the systematic registry were asthenia (93.6%), pain (80.7%), anorexia (78.9%), anxiety (69.5%), sadness (69.4%) and insomnia (60.6%). Two hundred seventy patients (44.5%) fulfilled the criteria of high symptom burden (greater than five chronic symptoms of at least moderate intensity) and 341 patients (56.2%) reported pain with potentially refractory characteristics according to the ECSCP. Significant functional impairment (Karnofsky index ≤ 60%) was observed in 264 patients (43.5%). According to the variables described in the PALCOM scale, 405 patients (66.7%) presented at least one factor of socio-familial risk, and 126 (20.7%) had at least some existential/ spiritual/ ethical conflicts. Three hundred seventy- nine patients (62.4%) died before completing the 6 months of follow-up. There were no significant differences in the socio-demographic data, primary origin of the cancer, symptom frequency, domains of the PALCOM scale or mortality at 6 months between the development and validation cohorts. The data are shown in Table 2.

In the pooled data, 118 (19.5%) patients were classified as having low complexity of PC needs according to the PALCOM scale, while 306 (50.5%) were classified as having medium and 182 (30.0%) high complexity (Table 2 and Table 3). No statistically significant differences were observed in the assignment of the level of complexity between the development and validation cohorts. In the pooled data of the two cohorts stratified by the level of complexity of the PALCOM scale, the following were observed: (a) significantly higher prevalence of high symptom burden in high levels of complexity (low 22.0%, medium 59.4%, high 89.0%) (*p* < 0.001); (b) significantly higher prevalence of prevalence of potentially refractory pain, according to the ECSCP, in high levels of complexity (low 36.4%, medium 52.9%, high 74.7%) (*p* < 0.001); (c) significantly greater prevalence of functional impairment (Karnoksky index ≤60%) in high levels of complexity (low 17.8%, medium 46.7%, high 54.9%) (*p* < 0.001); (d) significantly greater prevalence of at least one factor of socio-familial risk in high levels of complexity (low 42.5%, medium 67.6%, high 83.5%) (*p* < 0.001); (e) significantly greater prevalence of at least one ethical/existential/spiritual conflict in high levels of complexity (low 4.2%, medium 19.5%, high 30.8%) (*p* < 0.001); (f) significantly greater probability of death during the 6 months of follow-up in high levels of complexity (low 43.2%, medium 62.7%, high 75.8%) (*p* < 0.001); (g) significantly greater probability of hospital death in high levels of complexity (low 7.6%, medium 16.0%, high 28.6%) (*p* < 0.001). The data are shown in Table 3.

The comparison of the two cohorts at different times and by different teams allowed confirmation of the consistency of the PALCOM model based on: (a) the homogeneity of the socio-demographic and clinical data; (b) the lack of significant differences in the distribution of the frequencies of the levels of complexity; (c) the highly significant differences in the frequencies of the different domains of the scale according to the assigned level; and (d) the highly significant differences in 6-month mortality according to the assigned level.

### 3.2. Evolutionary Behavior of the Levels of Complexity (Table 4)

As mentioned previously, the analysis of the evolutionary behavior of the levels of complexity was only based on the 283 patients included in the validation cohort. In this section, the following variables are analyzed according to the level of complexity assigned to each patient at the basal visit: (1) instability ratio (IR), defined as the monthly probability of a change in the level of complexity or death; (2) monthly mean of the episodes of care in the emergency department; (3) monthly mean number of days of hospitalization; (4) survival.

**Table 4:** Evolution of complexity level, monthly probability of level change or death (instability ratio) and use of healthcare resources.

	Low				Medium				High				
	N (%)	Instability ratio N (cumulative %) *	Emergency. Mean ±SD **	Hospital days Mean ±SD *	N (%)	Instability ratio N (cumulative %) *	Emergency. Mean ±SD **	Hospital days Mean ±SD *	N (%)	Instability ratio N (cumulative %) *	Emergency. Mean ±SD **	Hospital days Mean ±SD *	*p*
Baseline	67 (23.7)				167 (59.9)				49 (17.3)				
Month 1	64 (95.5)	3 (4.5)	0.17 ±0.4	1.0 ± 3.1	153 (91.6)	14 (8.4)	0.22 ± 0.4	1.5 ± 4.8	40 (81.6)	9 (18.4)	0.31 ± 0.5	2.5 ± 6.7	* <0.001 ** NSD
Month 2	57 (85.1)	7 (14.9)	0.27 ±0.6	2.0 ± 5.5	118 (70.7)	35 (29.3)	0.24 ± 0.5	1.9 ± 5.6	26 (53.1)	14 (46.9)	0.25 ± 0.5	1.97 ± 5.8	* <0.001 ** NSD
Month 3	51 (76.1)	6 (23.8)	0.20 ±0.4	2.2 ± 5.2	90 (53.9)	28 (46.1)	0.25 ± 0.5	2.0 ± 5.5	16 (32.7)	10 (67.3)	0.26 ± 0.6	2.09 ± 6.6	* <0.001 ** NSD
Month 4	45 (67.2)	6 (32.8)	0.18 ±0.4	1.5 ± 4.1	71 (42.5)	19 (57.5)	0.22 ± 0.4	2.1 ± 5.4	12 (24.5)	4 (75.5)	0.23 ± 0.4	4.38 ± 9.5	* <0.001 ** NSD
Month 5	36 (53.7)	9 (46.3)	0.10 ±0.3	1.3 ± 5.1	67 (40.1)	4 (59.9)	0.23 ± 0.5	2.0 ± 5.9	11 (22.4)	1 (77.6)	0.25 ± 0.6	2.92 ± 7.9	* <0.001 ** NSD
Month 6	33 (49.3)	3 (50.7)	0.14 ±0.3	1.1 ± 2.8	53 (31.7)	14 (68.3)	0.24 ± 0.5	1.2 ± 3.1	11 (22.4)	0 (77.6)	0.17 ± 0.4	5.25 ± 10.6	* <0.001 ** NSD
Overall 6 months		34 (50.7)	0.2 ±0.3	1.5 ± 4.0		114 (68.3)	0.2 ± 0.5	1.8 ± 5.0		38 (77.6)	0.25 ± 0.5	3.2 ± 7.8	* <0.001 ** NSD

* Significant differences in probability of level change and mean hospitalization days per month among levels of complexity of palliative care needs. ** NSD: No significant differences in means of frequency of emergency department visits per month among levels of complexity of palliative care needs. SD: standard deviation.

#### 3.2.1. Instability Ratio (IR) According to the Level of PALCOM Complexity

**Figure 2:**
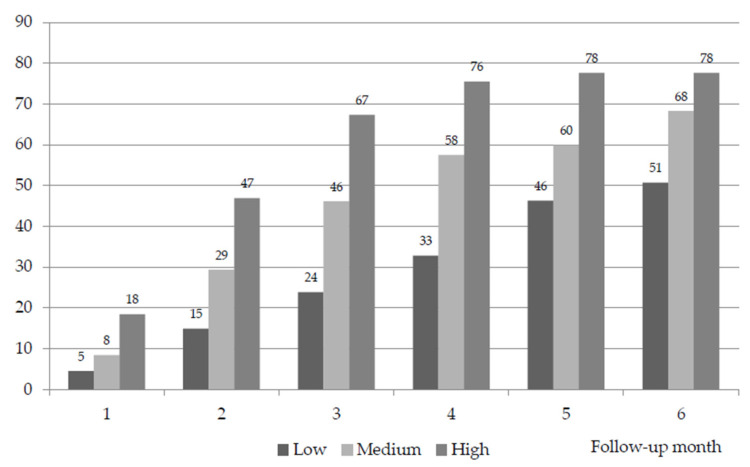
Cumulative instability rate over follow-up (monthly probability of level change or death) (%). Significant differences in monthly IR by level of palliative complexity during follow-up (*p* < 0.001).

The IR of the patients classified as low complexity in the basal visit was 4.5% in the first month of follow-up, 23.8% before the third month and 50.7% before the sixth month. The IR of the patients classified as having medium complexity in the basal visit was 8.4% in the first month of follow-up, 46.1% before the third month and 68.3% before the sixth month. The IR of the patients classified as having high complexity in the basal visit was 18.4% in the first month of follow-up, 67.3% before the third month and 77.6% before the sixth month. The differences in IR among the different levels of complexity at 6 months of follow-up were highly significant (*p* < 0.001) (Table 4) (Figure 2). In the comparison of the IR between the cases of medium and low complexity, the odds ratio (OR) was 2.7 (95% CI: 1.48, 4.74), being 1.6 (95% CI 0.76, 3.39) between the cases of high and medium complexity and 4.3 (95% CI: 1.86, 9.73) between those of high and low complexity.

A fundamental characteristic of the construct of complexity is that it depends on the interaction of multiple variables in an unstable equilibrium that is very sensitive to the changes in the conditions at initiation. The data observed in this study confirm that the levels of greatest complexity are significantly associated with greater instability, identified by the monthly probability of change in level or death.

#### 3.2.2. Indicators of Resource Consumption according to the PALCOM Level of Complexity

The mean frequency of emergency department services by the patients classified as low or medium complexity was 0.20 (SD ± 0.3) episodes per month, while that of patients considered to have high complexity was 0.25 (SD ± 0.5) episodes (no significant differences). The mean number of days of hospitalization in patients classified as low complexity was 1.5 (SD ± 4) days per month, 1.8 (SD ±5)) and 3.2 (SD ±7.8) days in those classified with medium and high complexity, respectively (*p* < 0.001).

In polled data of the two cohorts, among the 51 patients classified with low complexity who died during follow-up, nine (17.6%) died in the hospital. Of the 190 patients considered as medium complexity who died during follow-up, 49 (25.8%) died in hospital, and of the 138 patients classified as high complexity who died during follow-up, death occurred in hospital in 52 (37.7%). The differences in the probability of hospital death among the different levels of complexity were significant (*p* < 0.001).

From a general point of view, it can be concluded that the patients of the different levels of PC complexity presented a very similar probability of medical complications requiring emergency department care, but the impact on hospital stays and death in the hospital was significantly higher in those with higher levels of complexity.

#### 3.2.3. Survival according to the PALCOM Level of Complexity

**Figure 3:**
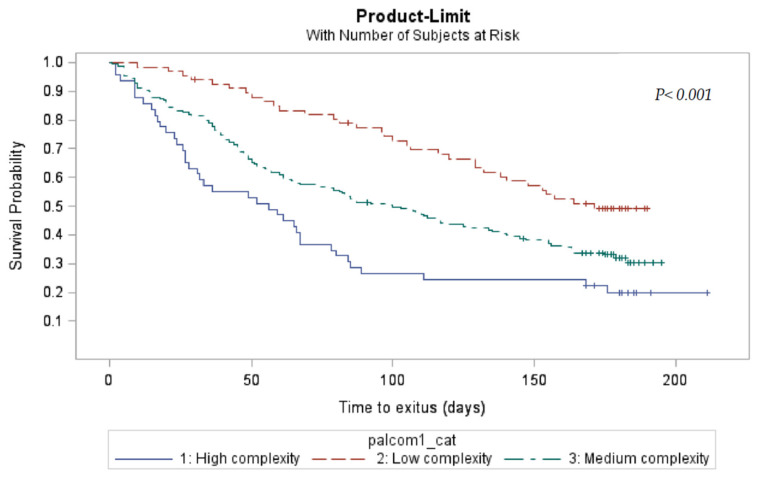
Actuarial survival by PALCOM level of complexity of palliative care needs (Kaplan–Meier).

Of the 607 patients included in the PALCOM development and validation cohorts, 228 (37.6%) remained alive at the final visit of the study. The 6-month survival of the 118 cases classified as having low PC complexity was 56.8%, being 37.9% in the 306 patients with medium complexity and 24.2% in the 182 cases of high complexity (*p* < 0.001). According to the Kaplan–Meier method, the monthly probability of survival was significantly greater in the levels of lower complexity (*p* < 0.001) (Figure 3).

### 3.3. Profiles of PALCOM Complexity

An integrated view of the data of this study allowed the description of generic profiles of the complexity of PC needs (Table 5).

#### 3.3.1. Profile of Low Complexity

(a) involvement of one or no domain of the scale PALCOM; (b) probability of a radical change from the initial conditions (increase in level or death) initially low (<15%), moderate between the third and sixth month of follow-up (25–45%); (c) frequency of visits to the emergency department similar to the other levels (mean: 0.2 episodes/month); (d) significantly lower hospital stay than the higher levels of complexity (mean: 1.2 days/patient/month); (e) survival significantly greater in relation to the other PALCOM levels (57% at 6 months of follow-up).

#### 3.3.2. Profile of Medium Complexity

(a) The involvement of two or three domains of the PALCOM scale; (b) probability of a radical change from the initial conditions (change in level of death) at least moderate initially (8–45%) and high between the third and sixth month of follow-up (57–68%); (c) frequency of visits to the emergency department similar to the other levels (mean: 0.2 episodes/month); (d) number of days of hospital stay significantly greater than that observed in patients with low complexity (mean: 1.8 days/patient/month); (e) survival significantly lower than that observed in low complexity and significantly higher that of patients with high complexity (38% at 6 months of follow-up).

#### 3.3.3. Profile of High Complexity:

(a) The involvement of four or five domains of the PALCOM scale; (b) probability of a radical change from the initial conditions (change in level of death) initially high (19–67%) and very high between the third and sixth month of follow-up (>75%); (c) frequency of visits to the emergency department similar to the other levels (mean: 0.2 episodes/month); (d) number of days of hospital stay significantly greater than that observed in patients with lower levels of complexity (mean: 3.2 days/patient/month); (e) survival significantly lower than that observed in the levels of low and medium complexity (24% at 6 months of follow-up).

## 4. Discussion

In the pooled analysis, the distribution of the frequencies of the levels of complexity between the two cohorts was very homogeneous and the differences in the frequencies of the domains of the PALCOM scale stratified by the level of complexity assigned were highly significant. These results confirm the consistency of the PALCOM model of classification of the complexity of PC needs, also considering that they were obtained in two cohorts studied in different periods of time and by different teams.

In the evolutionary analysis of a maximum follow-up of 6 months, the complexity was significantly associated with a greater monthly probability of change in the level of complexity or death (IR), healthcare resource consumption and hospital death as well as a lower survival, all of which confirm the primary hypothesis of this study.

The PALCOM scale also has a high predictive value for mortality in the short and medium term, although it was not designed for this purpose. In this context, a structured assessment of palliative complexity and estimation of survival, as identified by the PALCOM scale, should facilitate supportive planning, decision-making, and the use of healthcare resources focused on the essential needs of patients.

According to the systemic theory, the complexity of PC needs is characterized by the continuous adaptative interaction of multiple parts in a not-always-linear relationship and in unstable equilibrium. The instability of the system is related to the high sensitivity to the frequent and rapid changes of the initial conditions, the result of which cannot always be predicted and may be different and greater than the sum of the parts implicated [29,30,31,52,53,54]. The complexity of needs in the end-of-life process depends not only on the characteristics of the vital multidimensional experiences of each individual but also on the conditions of the social environment and those of support [30,52,53,54]. The data of the development and validation cohorts of the PALCOM scale, individually and all together, as well as the analysis of their evolutionary behavior, adjust to the construct of complexity proposed by the systemic theory. First, it can be seen that the grade of complexity assigned by the PALCOM scale depends more on the interaction of multidimensional variables than on the intensity of one variable alone. Second, the PALCOM scale describes an unstable dynamic, in that the complexity is associated with a greater probability of change in level or death (IR). Finally, the PALCOM scale evaluates the interaction between the vital experiences of the individual with their care environment, allows for the identification of priority areas of care and describes profiles of complexity that help manage the shared intervention of the specialized EPC resources (primary, secondary or tertiary PC).

In a context in which healthcare resources are not unlimited, a controversial point is the model of referral to EPC (systematic, on-demand or targeted). At present, many experts consider that the targeted referral model based on systematic evaluation of the specific needs of each patient is the most appropriate [26,27,28]. Targeted referral is based on criteria of consensus among the referent teams and of EPC, the goal of which is to ensure that all patients receive PC that is adapted to their needs and avoid unnecessary activation of specialized PC resources in cases of low complexity. One indispensable condition of the targeted referral model is that the reference healthcare teams must maintain and promote the basic competencies of PC. Some authors classify basic PC as primary or secondary and specialized PC as tertiary [25]. Primary PC is established within the community setting by Primary Care, in which basic PC is added to the competencies of advanced chronicity inherent in the specialty [55,56]. Secondary PC is provided by specialized professionals, which in this case are from Oncology, in which basic PC is added to the advanced competencies in the evolutionary complications of cancer and the toxicity of the treatments [16,57,58]. Lastly, tertiary PC is provided by multidisciplinary teams specialized in EPC that support the Primary Care and specialized teams in a model of shared care focused on cases with greater needs [26,27,28,59,60,61,62]. It is reasonable to think that the identification of patient profiles based on PC complexity may be of great utility as a criterion of targeted referral and for the management of the intensity of care shared among the referent and specialized teams.

Several tools that define and categorize the construct of the complexity of PC needs have been described (Hex-Com, Perroca-Scale, AN-SNAP, Hui–Major criteria, IDC-Pal, PALCOM) [29,30,31,32,33,34,35,36,37,38,39,40,41,42,43,44,45,46]. All these tools include a multidimensional evaluation of the patient with the aim of establishing early referral criteria for multidisciplinary teams specialized in EPC. In some cases, the methodology of development of these scales is based on the consensus of experts regarding the conditions of complexity and their validation in transversal or Delphi studies [32,33,34], and in other cases it is based on prospective observational studies to determine predictive variables of complexity and their external validations (PALCOM scale) [31,46].

### Study Limitations

The PALCOM scale was developed for adult patients with cancer. Therefore, the usefulness of this tool in pediatric patients or those with advanced chronic non-cancer diseases cannot be confirmed without specific studies.

This study was carried out within a wide public healthcare network with universal access to all citizens, and in which PC is included among the services provided. It is unclear whether the PALCOM scale can be validated in different healthcare settings.

The development and validation cohorts of the PALCOM scale included patients with advanced cancer and an estimated life expectancy of less than 6 months. Although it is reasonable to assume that the PALCOM scale may be consistent in patients with longer estimated survival, further studies are needed in this regard.

Although the domain of the PALCOM scale related to ethical/existential/ spiritual conflicts may depend on the communication skills or the subjectivity of the professional evaluator, the results of the study do not confirm this potential limitation since no significant differences were observed in the registry of this domain between the two cohorts carried out in different periods of time and by different teams.

## 5. Conclusions

The results of this study confirm the consistency and high discriminative capacity of the PALCOM scale to establish the level of complexity of palliative care needs.

The complexity of the palliative care needs to be classified by the PALCOM scale was significantly associated with unstable equilibrium, an increase in the consumption of healthcare resources, a lower survival and a greater probability of death in the hospital.

The analysis of the domains of the PALCOM scale and their evolutionary behavior allows the description of patient profiles according to their complexity and the management of not only early referral to multidisciplinary teams specialized in EPC but also the intensity of their intervention in a model of care shared with the referent services.

### 5.1. Practical Implications

In integrated care based on the essential needs of the patient, it is essential to have validated tools to identify the complexity of care. The systematic use of the PALCOM scale within the healthcare setting of patients with advanced cancer can optimize targeted referral to EPC so that no patient misses the opportunity of receiving adequate palliative care, patients with greater needs are prioritized, and unnecessary specialized resources are not systematically activated in situations of low complexity.

The reinforcement of the conceptualization of non-specialized palliative care (primary and secondary palliative care), the promotion of basic training in palliative care of the referring teams and the establishment of criteria of targeted referral to teams specialized in EPC (tertiary palliative care) based on the complexity of palliative care needs, would undoubtedly contribute to improving the care of patients with cancer in the end of life process.

### 5.2. Implications for Investigation

We believe that the PALCOM scale represents a significant advance in integrated palliative care of patients with cancer. However, it is necessary to widen the investigation not only to determine the global prevalence of the levels of complexity from the diagnosis of advanced disease but also to determine the impact on the management of shared care and on health results.

Since this study was based on patients with life expectancies of less than 6 months, in order to know the real prevalence of the levels of complexity in patients with advanced cancer, a transversal study from the time of the diagnosis of advanced disease is necessary. In this case, the prevalence of low palliative complexity would likely be higher than that observed in the present study because less evolved patients with a greater survival time would be included. Nonetheless, the clinical evolutionary behavior of the patients with medium-high palliative needs complexity would likely be similar.

To minimize the potential variability in the registry of the scale, it would be of interest to carry out the studies necessary to transform the PALCOM scale into a structured tool self-administered by the patients or by non-specialized professionals. In this regard, a qualitative study including patients (focus group) aimed at constructing a questionnaire in the form of questions and answers adjusted to the criteria of the PALCOM scale, and that is easily understood and does not make patients uncomfortable, should be carried out. Afterward, a validation study of this self-administered tool would be necessary.

## Figures and Tables

**Table 1:** PALCOM—Palliative Care Needs Complexity Scale [31,46].

**First, surprise question:** Would I be surprised if this patient died in the next 12 months? If the answer is no, the PALCOM scale can help you determine the level of complexity of palliative care needs and manage the intensity of specialist palliative care team intervention in a model of early shared care.	
**Second, explore PALCOM domains:** The level of palliative complexity can be categorized by assessing the following five domains.	
**Domain 1:** Symptom burden	**Is a high symptom burden detected?** Assess at least the following chronic symptoms: pain; anorexia; weakness; nausea-vomiting; constipation; dyspnoea or cough; insomnia; drowsiness; anxiety; sadness; others.A high symptom burden is considered to exist if the patient experiences ≥5 of these or other chronic symptoms with an intensity of at least moderate on a categorical scale, or ≥4 on a numerical or visual analogue scale of 10 points.
**Domain 2:** Markers of difficult pain	**Are there any markers of difficult pain control?** Any of the following conditions can cause potentially difficult pain: neuropathic pain; mixed pain (nociceptive and neuropathic); breakthrough cancer pain; pain associated with a history of alcohol or other substance abuse, or cognitive impairment or severe emotional distress.
**Domain 3:** Functional status	**Is there functional impairment?** Patients who require significant assistance with activities of daily living. (e.g., Barthel Index ≤60 or Karnofsky Index ≤50–60%)
**Domain 4:** Socio-familial risk	**Any socio-familial risk factors?** Any of the following conditions may be considered a socio-family risk factor: absence of identified caregiver; caregiver limitations due to advanced age, health problems, or socio-family or economic burdens; minors or more than one member of the nuclear family who needs support; risk of severe family burnout; other complexity situations (social vulnerability, poverty, domestic violence, addiction of abuse substances, etc.)
**Domain 5:** Spiritual/existential/ethic problems	Any ethical or existential conflict? Any of the following conditions may be considered: conflicts related to information (denial, conspiracy silence, ...); healthcare team disagreement; disagreement between patient/family and healthcare team; loss of meaning in life or existential distress; spiritual distress; desire to advance death, demand for euthanasia or assisted suicide; others.
**Third, scoring:** Each of these five domains is scored dichotomously (0 for the absence and 1 for the presence of any variable in each domain). The sum of the scores between 0 and 5 is the total score of the PALCOM scale.	
**Fourth, determine the level of palliative complexity according to the observed score:** ▪ **0–1 Low complexity:** Basic palliative care is recommended. Referring team to get back in contact if patient becomes more complex. In some cases, timely consultation with specialist palliative care may be needed for a comprehensive assessment or management of difficult isolated symptoms. ▪ **2–3 Medium complexity:** Specialized palliative care is systematically recommended (hospital teams, home support teams or palliative care services). ▪ **4–5 High complexity:** Intensive specialized palliative care is systematically recommended (teams in the hospital, support teams in the home or palliative care services).	

**Table 2:** Characteristics of patients according to the development and validation cohorts of the PALCOM scale and pooled data.

		Development Cohort	Validation Cohort		PALCOM Pooled Data
		N (%)	N (%)	*p*	N (%)
Total		324	283		607
Gender	Male	189 (58.0)	161 (56.9)	0.280	350 (57.7)
Age	(mean ± SD *)	69 (SD ± 59–80)	71 (SD ± 59–81)	0.320	70 SD ± 59–80
Primary origin					
	Lung	71 (21.9)	64 (24.4)	0.310	140 (23.1)
	Colon	38 (11.7)	56 (19.8)	0.290	94 (15.5)
	Pancreas	28 (8.6)	18 (6.4)	0.270	46 (7.6)
	Breast	22 (6.8)	19 (6.7)	0.190	41 (6.8)
	Prostate	18 (5.5)	28 (9.9)	0.210	46 (7.6)
	Others	146 (45.1)	93 (32.8)	0.300	239 (39.4)
Symptom prevalence					
	Asthenia	299 (92.2)	269 (95.1)	0.210	568 (93.6)
	Anorexia	253 (78.1)	226 (79.9)	0.079	479 (78.9)
	Pain	245 (75.6)	245 (86.6)	0.100	490 (80.7)
	Nausea	110 (34.0)	68 (24.0)	0.080	178 (29.3)
	Constipation	202 (62.3)	162 (57.2)	0.110	364 (59.9)
	Dyspnoea	149 (45.9)	111 (39.2)	0.220	260 (42.8)
	Insomnia	191 (58.9)	177 (62.5)	0.180	368 (60.6)
	Anxiety	238 (73.4)	184 (65.0)	0.090	422 (69.5)
	Sadness	225 (69.4)	196 (69.3)	0.220	421 (69.4)
PALCOM domains					
	High symptom burden	134 (41.3)	136 (48.1)	0.190	270 (44.5)
	Refractory pain	175 (54.0)	166 (58.5)	0.220	341 (56.2)
	Karnoksky index ≤ 60%	129 (39.9)	135 (47.5)	0.210	264 (43.5)
	Socio-familial risk	221 (68.3)	184 (64.8)	0.420	405 (66.7)
	Existential/ethical conflicts	59 (18.3)	67 (23.6)	0.080	126 (20.7)
PALCOM scale level					
	Low	51 (15.8)	67 (23.7)	0.080	118 (19.5)
	Medium	139 (43.0)	167 (59.9)	0.780	306 (50.5)
	High	133 (41.2)	49 (17.3)	0.060	182 (30.0)

* standard deviation.

**Table 3:** PALCOM scale domains according to the level of complexity of palliative care needs.

		Development Cohort				Validation Cohort				Pooled Data			
N (%)		324 (53.4% of polled data)				283 (46.6% of polled data)				607			
Hospital inclusion		180 (55.6)					175 (61.8)				355 (58.5)		
Community inclusion		144 (44.4)					108 (38.2)				252 (41.5)		
		Low	Medium	High		Low	Medium	High		Low	Medium	High	
		51 (15.8)	139 (43.0)	133 (41.2)		67 (23.7)	167 (59.9)	49 (17.3)		118 (19.5)	306 (50.5)	182 (30.0)	
PALCOM domains					*p*				*p*				*p*
	High symptom burden	20 (39.2)	99 (71.2)	115 (86.5)	<0.001	6 (8.9)	83 (49.7)	47 (95.5)	<0.001	26 (22.0)	182 (59.4)	162 (89.0)	<0.001
	Refractory pain	13 (25.5)	69 (49.6)	93 (69.9)	<0.001	30 (45.7)	93 (55.4)	43 (87.8)	<0.001	43 (36.4)	162 (52.9)	136 (74.7)	<0.001
	Karnofsky index <60%	17 (33.3)	53 (38.1)	59 (44.4)	<0.001	4 (5.9)	90 (53.6)	41 (83.7)	<0.001	21 (17.8)	143 (46.7)	100 (54.9)	<0.001
	Socio-familial risk	33 (64.7)	81 (58.3)	107 (80.5)	0.055	13 (19.4)	126 (75.0)	45 (91.8)	<0.001	46 (42.5)	207 (67.6)	152 (83.5)	<0.001
	Existential/ethical conflicts	1 (2.0)	25 (18.0)	33 (24.8)	<0.001	4 (5.9)	34 (20.2)	29 (59.2)	<0.001	5 (4.2)	59 (19.5)	56 (30.8)	<0.001
Death within 6 months		18 (35.3)	77 (55.4)	99 (74.3)	<0.001	33 (49.2)	114 (68.3)	38 (77.6)	<0.001	51 (43.2)	190 (62.7)	138 (75.8)	<0.001
Hospital death		3 (5.9)	24 (17.3)	40 (30.1)	<0.001	6 (8.9)	25 (22.1)	12 (30.8)	<0.001	9 (7.6)	49 (16.0)	52 (28.6)	<0.001

**Table 5:** Profiles of complexity of palliative care needs in cancer patients.

Level	Characteristics
Low	Involvement of 0 or 1 domains on the PALCOM scale. Low likelihood of radical change in baseline conditions in the first 3 months, at least moderate in next months. Frequency of emergency department visits similar to other levels (cancer complications or treatment toxicity). Need for hospitalization significantly lower than other levelsSurvival significantly higher than other levels.
	▪ PALCOM 0: Palliative care by non-specialized referral teams (primary PC ** or secondary PC ***) is recommended. ▪ PALCOM 1: Palliative care by non-specialized referral teams (primary PC or secondary PC) and timely consultations in difficult-to-control conditions to specialized multidisciplinary EPC teams (tertiary care ****) is recommended. ▪ In a stable situation, reassess the level of complexity at intervals < 2–3 months.
Medium	Involvement of 2–3 domains on the PALCOM scale. Low-moderate likelihood of radical change in baseline conditions in the first 3 months, at moderate-high in next months. Frequency of emergency department visits similar to other levels (cancer complications or treatment toxicity). Need for hospitalization significantly higher than low complexity level. Survival significantly lower than low complexity level.
	▪ Systematic shared care between referring professionals and specialized multidisciplinary EPC teams (outpatients, hospital or home teams) (tertiary care) is recommended.
High	Involvement of 4–5 domains on the PALCOM scale. At least moderate likelihood of radical change in baseline conditions in the first weeks, at very high since second month of follow-up. Frequency of emergency department visits similar to other levels (cancer complications or treatment toxicity). Need for hospitalization significantly higher than other levels Survival significantly lower than other levels.
	▪ Systematic intensive shared care between referring professionals and specialized multidisciplinary EPC teams (outpatients, hospital or home teams) (tertiary care) is recommended.

Categorization of frequencies in this context: low < 20%; moderate 21–40%; high 41–60%; very high >60%. ** Primary PC: Primary Care professionals and basic PC training. *** Secondary PC: Professionals specialized in oncology and basic training in PC. **** Tertiary PC: Professionals specialized in EPC. PC: palliative care. EPC: early palliative care.

## Data Availability

The data can be shared upon request.
